# Fasting induces anti-Warburg effect that increases respiration but reduces ATP-synthesis to promote apoptosis in colon cancer models

**DOI:** 10.18632/oncotarget.3688

**Published:** 2015-03-18

**Authors:** Giovanna Bianchi, Roberto Martella, Silvia Ravera, Cecilia Marini, Selene Capitanio, Annamaria Orengo, Laura Emionite, Chiara Lavarello, Adriana Amaro, Andrea Petretto, Ulrich Pfeffer, Gianmario Sambuceti, Vito Pistoia, Lizzia Raffaghello, Valter D. Longo

**Affiliations:** ^1^ Laboratorio di Oncologia Istituto G. Gaslini, Genoa, Italy; ^2^ Department of Pharmacy, University of Genoa, Genova, Italy; ^3^ CNR Institute of Bioimages and Molecular Physiology, Milan, Section of Genoa, Genoa, Italy; ^4^ Nuclear Medicine Unit, Department of Health Sciences, University of Genoa and IRCCS AOU San Martino - IST Istituto Nazionale per la Ricerca sul Cancro, Genoa, Italy; ^5^ Animal facility, IRCCS AOU San Martino - IST Istituto Nazionale per la Ricerca sul Cancro, Genoa, Italy; ^6^ Core Facility, Istituto G. Gaslini, Genoa, Italy; ^7^ Functional Genomics, IRCCS AOU San Martino - IST Istituto Nazionale per la Ricerca sul Cancro, Genoa, Italy; ^8^ Longevity Institute, School of Gerontology, Department of Biological Sciences, University of Southern California, Los Angeles, CA, USA; ^9^ Eli and Edythe Broad Center for Regenerative Medicine and Stem Cell Research at USC, Keck School of Medicine, University of Southern California, Los Angeles, CA, USA; ^10^ IFOM, FIRC Institute of Molecular Oncology, Milan, Italy

**Keywords:** Fasting, Warburg effect, colon cancer, oxidative phosphorylation, glucose uptake

## Abstract

Tumor chemoresistance is associated with high aerobic glycolysis rates and reduced oxidative phosphorylation, a phenomenon called “Warburg effect” whose reversal could impair the ability of a wide range of cancer cells to survive in the presence or absence of chemotherapy. In previous studies, Short-term-starvation (STS) was shown to protect normal cells and organs but to sensitize different cancer cell types to chemotherapy but the mechanisms responsible for these effects are poorly understood. We tested the cytotoxicity of Oxaliplatin (OXP) combined with a 48hour STS on the progression of CT26 colorectal tumors. STS potentiated the effects of OXP on the suppression of colon carcinoma growth and glucose uptake in both *in vitro* and *in vivo* models. In CT26 cells, STS down-regulated aerobic glycolysis, and glutaminolysis, while increasing oxidative phosphorylation. The STS-dependent increase in both Complex I and Complex II-dependent O_2_ consumption was associated with increased oxidative stress and reduced ATP synthesis. Chemotherapy caused additional toxicity, which was associated with increased succinate/Complex II-dependent O_2_ consumption, elevated oxidative stress and apoptosis.

These findings indicate that the glucose and amino acid deficiency conditions imposed by STS promote an anti-Warburg effect characterized by increased oxygen consumption but failure to generate ATP, resulting in oxidative damage and apoptosis.

## INTRODUCTION

Tumor cells are characterized by high glucose uptake and lactate production regardless of oxygen concentration, a phenomenon known as the “Warburg effect” [1]. The metabolic shift toward the Warburg effect confers bioenergetic and biosynthetic advantages to proliferating cells by increasing non-oxidative ATP production and generating metabolic intermediates from glucose important for cell growth [2]. Glutamine represents an additional metabolite catabolized by tumor cells and utilized for biosynthetic processes [3]. Aerobic glycolysis and glutaminolysis contribute to the chemoresistance of cancer cells [4] through :i) increased lactate production and export, ii) elevated NADPH limiting DNA oxidant drug effectiveness, and iii) decreased oxidative phosphorylation (OXPHOS) rate leading to reduced reactive oxygen species (ROS)-mediated DNA damage [5-8]. Therefore, strategies inhibiting glycolysis and glutaminolysis and promoting OXPHOS in order to delay tumor growth and overcome drug resistance are being investigated [9-11]. The return of cancer cells to the normal respiratory mode maintained by normal cells could also promote cancer cell death if the respiration was accompanied by electron leakage and superoxide generation [12, 13], leading to cellular damage and apoptosis [14, 15].

In mice, three day cycles of water only fasting (Short-Term-Starvation, STS) cause a generalized glucose and amino acid reduction/deficiency, which protects normal but not tumor cells against chemotherapy-mediated cytotoxicity [16], and induces potent chemosensitizing effects in many tumors [17, 18].

Preliminary clinical data indicates that in cancer patients fasting is not associated with major side effects and may reduce several of the side effects associated to chemotherapy [19] including a decreased toxicity to lymphocytes [20]. These pre-clinical and preliminary clinical results served as the foundation for randomized trials to test the safety and efficacy of fasting cycles on the effect of chemotherapy on both normal and cancer cells (NCT01304251, NCT01175837, NCT00936364, NCT01175837).

However, the molecular mechanisms responsible for the chemosensitizing effects of fasting on cancer cells remain poorly understood [17, 21].

Here, using both *in vitro* and *in vivo* colon carcinoma models, we show that STS exerts an anti-Warburg effect driving tumor cells from a glycolytic mode into an uncoupled OXPHOS which promotes increased ROS generation and apoptosis. These effects are enhanced by chemotherapy treatment.

## RESULTS

### Effects of fasting cycles and chemotherapy on colon carcinoma growth and glucose consumption *in vivo*

**Figure 1:**
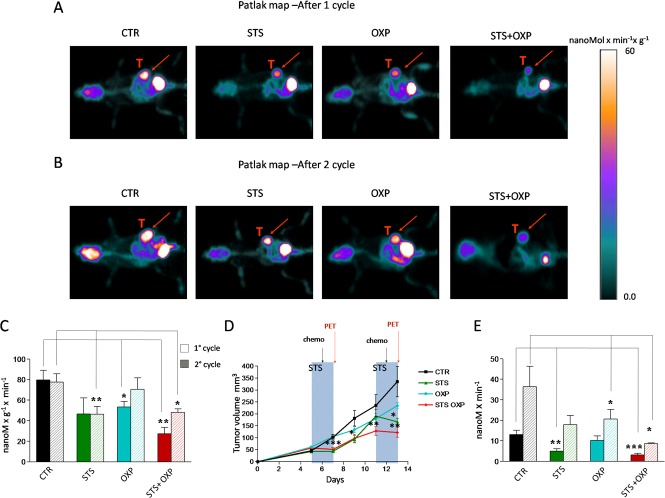
*In vivo* effect of fasting cycles in combination with chemotherapy on tumor glucose consumption and cancer growth CT26 cells were subcutaneously inoculated in the fat pad of BALB/c mice (200.000 cells/mouse). Five days after tumor cell inoculum, the mice were either fasted or maintained on the ad lib standard diet for 48 hours and treated with Oxaliplatin (OXP) (10 mg/Kg). After 1 week, the treatment was repeated. All mice were imaged after the first and the second cycle of therapy by a dedicated micro-PET system. Panel A shows the Patlak-map of a representative mouse for each group after the first cycle of treatment. Panel B shows the Patlak-map of a representative mouse for each group after the second cycle of treatment. Red arrows indicate the tumor mass. Panel C shows the cancer average glucose consumption expressed as nMol x min^−1^ x gr^−1^. Panel D shows the tumor volume expressed as mean value ± SD. Groups of experiments include: control (black), STS (green), OXP (light blue), and STS+OXP (red). Panel E shows the total cancer glucose consumption expressed as nMol x min^−1^.

We investigated the effect of STS +/− chemotherapy on glucose metabolism in CT26 colon carcinoma cells in mice by micro-PET analyses. As previously described [16, 17], 48-hour of STS induced a significant body weight loss and serum glucose reduction. Analysis of tracer uptake by the tumor showed a differential response to the treatments (Figure 1A-C). After the first cycle, STS was as effective as oxaliplatin (OXP) in reducing the average tumor glucose consumption (Figure 1C). However, the lowest values were achieved by STS+OXP (Figure 1A-C). Monitoring of tumor progression during multiple cycles revealed that average tumor metabolism was inhibited similarly by the first and second STS treatments (Figure 1C). Chemotherapy induced only a transient reduction of the lesion metabolic rate after its first application. By contrast, average glucose consumption remained significantly lower in STS+OXP-treated mice (Figure 1C).

The metabolic response to treatment was paralleled by an evident effect of STS on cancer growth, mostly during the fasting and not the post-fasting period (Figure 1D). The transient effect of STS on tumor growth was repeatable. OXP instead showed a deceleration in cancer growth which was enhanced by STS (STS+OXP) (Figure 1D). The additive effect of STS+OXP was also evident when total cancer glucose consumption rate was measured (tumor glucose metabolism/gr/min x total tumor volume). After both cycles, this glucose consumption rate was much lower in either STS- or OXP-treated mice but was lowest in STS+OXP-treated mice compared to that in untreated mice (STS+OXP *vs* STS 1° cycle P=0.05; STS+OXP *vs* OXP 1° cycle P=0.03; STS+OXP *vs* OXP 2° cycle P=0.01) (Figure 1E). In summary, these results indicate that STS enhances the toxicity of chemotherapy to colon cancer cells, resulting in decreased glucose consumption rates.

### *In vitro* effects of STS and chemotherapy on viability and metabolism of colon carcinoma cells

**Figure 2:**
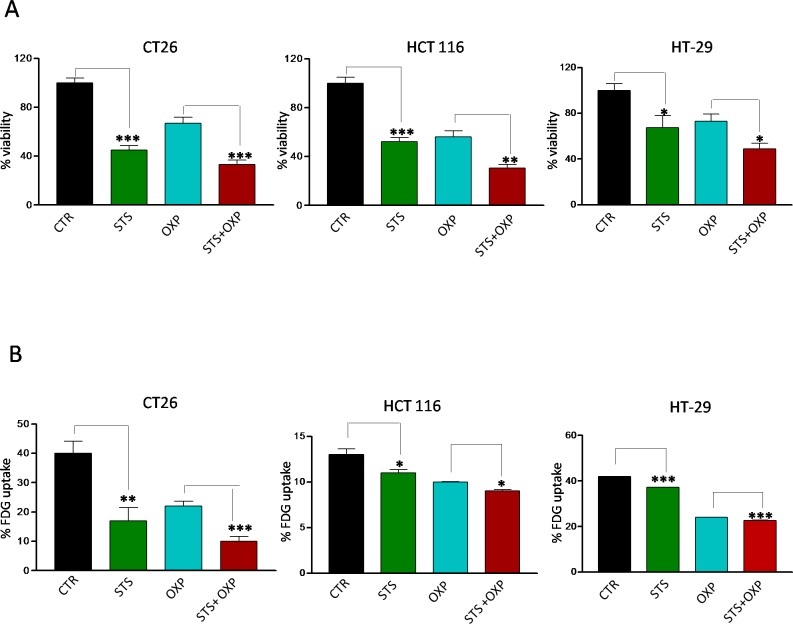
Effects of STS in combination with chemotherapy on viability and glucose uptake by colon carcinoma cells Tumor cells were cultured in with either low glucose (0.5 g/l) and 1% serum (*in vitro* STS) or the standard glucose levels (1.0 g/l) and 10% serum (control) for 48 hours. Then, cells were incubated with 40 μM oxaliplatin (OXP) for 24 hours. Panel A shows cell viability of different mouse and human colon carcinoma cell lines (CT26, HCT 116 and HT-29) as determined by Trypan Blue Assay. Panel B shows 18F-Fluorodeoxyglucose (FDG) uptake by different colon carcinoma cells (CT26, HCT 116 and HT-29). Tumor cells were incubated with FDG at 37 KBq/ml for 60 minutes. FDG retention was measured as the ratio between bound and total radioactivity. Data are expressed as percentage of viable cells ± SD. P value was calculated using unpaired t-test with Welch's correction. *: P<0.05; **: P<0.01; ***: P<0.001.

We investigated the *in vitro* effects of STS on a panel of colon carcinoma cell lines grown under normal or conditions mimicking starvation [17] for 48 hours. One day after STS, the cells were treated with OXP. STS and OXP showed additive cytotoxic effects in all the cell lines tested (Figure 2A). FDG uptake paralleled viability response since it was reduced by a similar degree by each single stressor, although the greatest impairment occurred in response to STS+OXP (Figure 2B). These results confirm the *in vivo* results and support the use of the *in vitro* paradigm to model the effects of STS in mice.

### STS and chemotherapy differentially regulate proliferation and metabolic enzymes in colon carcinoma cells

**Figure 3:**
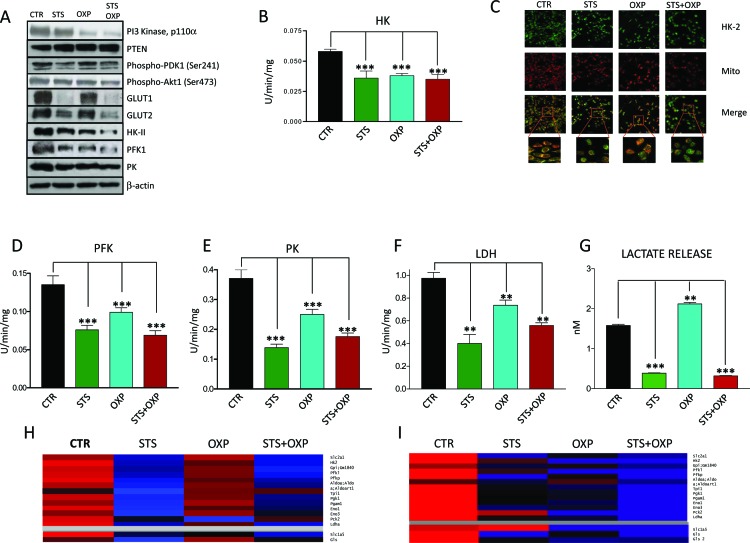
Effect of STS in combination with chemotherapy on growth and glucose metabolism pathways in colon carcinoma cells CT26 colon carcinoma cells, undergoing STS or incubated under standard conditions and treated with or without 40 μM oxaliplatin (OXP) for 24 hours, were lysed and underwent either western blots or enzymatic activity assays. For protein expression analysis cells were lysed and injected in a nanoscale high-performance liquid chromatography system connected to a hybrid linear trap quadrupole (LTQ) Orbitrap mass spectrometer. Proteins were identified and quantified using MaxQuant pipeline. For gene expression analysis RNA was extracted from CT26 cells and analyzed by microarray hybridization. Panel A shows the expression of PI3K, PTEN, Phospho-PDK1, Phospho-AKT, GLUT1, GLUT2, HKII, PFK1, PK and β-actin. Panel B shows the overall activity of HK by CT26 cells. Panel C shows the confocal microscopy for HK II. After treatment the cells were labeled by MitoTracker Far Red and stained for HK II expression by immunofluorescence. Merged images indicate that STS+OXP causes a significant and selective dislocation of HK II isoform from mitochondrial membrane to the cytosol. Panel D-E-F show the activity of Phosphofructokinase (PFK), Pyruvate kinase (PK), Lactate Dehydrogenase (LDH) by CT26 cells, respectively. Panel G shows the lactate release measured by spectrometry in the supernatants of CT26 cells. Data are expressed as mean values ± SD. P value was calculated using unpaired t test with Welch's correction. *: P<0.05; **: P<0.01; ***: P<0.001. Representative expression heat maps of proteins (Panel H) and genes (Panel I) involved in glycolysis and glutaminolysis are shown. Data are expressed as ratios over mean values for the four conditions (STS, OXP, STS+OXP; red = expression above mean, black= expression at mean; blue = expression below mean).

Because CT26 cells displayed the greatest sensitivity to STS in terms of viability and metabolic response (Figure 2A-B), we selected these cells to measure key mediators of proliferation and glucose metabolism in the PI3K-AKT pathway (PI3K, Phosphatase and Tensin Homolog (PTEN), 3-Phosphoinositide Dependent Kinase 1 (PDK1) and AKT). The effect of STS on metabolism was also evaluated by measuring GLUT1 and GLUT2 (both involved in glucose uptake) and the glycolytic enzyme HKII, PFK, Pyruvate Kinase (PK), and Lactate Dehydrogenase (LDH) expression and enzymatic activity. STS and in particular STS+OXP down-regulated the expression of PI3K/p110 (STS *vs* CTR: 81%; STS+OXP *vs* CTR: 48%), phospho-PDK1 (STS *vs* CTR: 57%; STS+OXP *vs* CTR: 84%) and phospho-AKT (STS *vs* CTR: 75%; STS+OXP *vs* CTR: 58%) and up-regulated PTEN expression (STS *vs* CTR: 122%; STS+OXP *vs* CTR: 121%) (Figure 3A). Notably, the latter effects matched well the effects of the treatments on CT26 proliferation (Supplementary Figure 1).

The effects of STS on proteins involved in glucose transport and metabolism was even more evident: STS induced a profound reduction in GLUT1 (STS *vs* CTR: 15%), GLUT2 (STS *vs* CTR: 40%), HKII (STS *vs* CTR: 74%; STS+OXP *vs* CTR: 48%), PFK1 (STS vs CTR: 74%; STS+OXP *vs* CTR: 48%), PK (STS *vs*CTR:64%;STS+OXP *vs* CTR: 66% ) protein expression, particularly in combination with chemotherapy (Figure 3A).

HK II catalytic function was reduced by all treatments (Figure 3B) but its intracellular localization was also affected. Immunofluorescence analysis showed a re-localization of HK II from the mitochondrial membrane into the cytosol, particularly after STS+OXP treatment (Figure 3C). Glucose metabolism impairment was further aggravated by the concomitant reduction of PFK (Figure 3D), PK (Figure 3E) and LDH (Figure 3F) activity caused by all treatments. STS-associated impairment of enzymes involved in glucose metabolism was confirmed by the analysis of extracellular lactate concentration, which was markedly reduced by STS alone or in combination with OXP (Figure 3G).

The STS-mediated reduction of glucose catabolism and expression of transport enzymes is consistent with the reduced levels of glucose caused by STS but also with the possibility that STS conditions promote a Warburg reversing effect.

### Expression of glycolytic and glutaminolytic enzymes in response to STS and chemotherapy

The transcriptome and proteome of STS- and OXP-treated CT26 cells were analyzed by oligonucleotide microarrays and Label Free Quantitation (LFQ) on High Resolution/Mass Accuracy Liquid Chromatography Tandem Mass Spectrometry (HR/MA LC MS/MS), respectively. Figure 3H shows a heat map of proteins involved in glycolysis and glutaminolysis. The down-regulation of glycolytic enzymes was observed in STS-treated CT26 cells (Figure 3H), with or without OXP treatment. This down-regulation is most likely due to an effect of STS at the transcriptional level since the expression profiles of STS-treated cells show a clear down-regulation of the genes encoding these enzymes (Figure 3I). At the gene expression level, OXP-treated cells also show reduced expression of glycolysis genes but this effect is more evident in STS+OXP-treated cells (Figure 3I). STS also reduced both glutaminase (Gls) mRNA and protein levels. In contrast, the expression of the glutamine transporter Slc1a5 was reduced only at the protein level (Figure 3H-I). The combination of STS+OXP reduced protein and mRNA levels of both glutaminase and the Slc5a1 (Figure 3H-I). These results confirm that the reduced glucose levels in combination with other changes imposed by STS, have a major effect in down-regulating glycolytic genes and proteins.

### Starvation and chemotherapy promote uncoupling of the mitochondrial respiratory chain and oxidative stress

**Figure 4:**
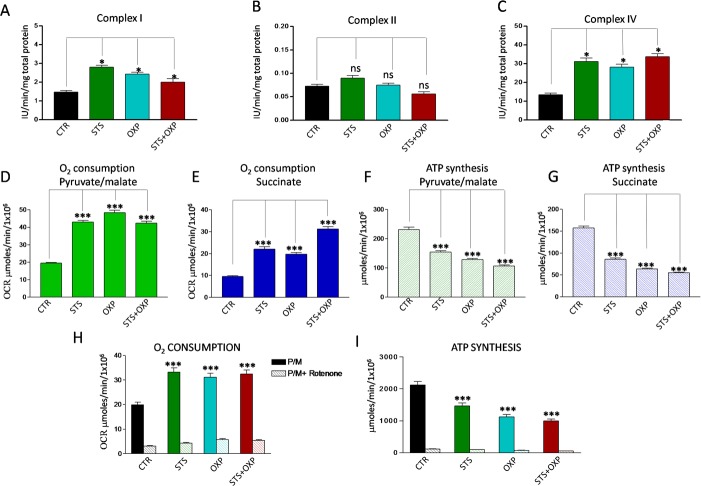
Effect of STS in combination with chemotherapy on oxidative phosphorylation activity of CT26 colon carcinoma cells Panels A, B and C report the activity of the redox Complexes I, II and IV in CT26 cell lines treated with STS +/− OXP. Data are expressed as mean values ± SD. P value was calculated using unpaired t-test with Welch's correction. *: P<0.05. Panels D-E show the oxygen consumption rate (OCR) of CT26 in the presence of pyruvate (10mM)/malate (5mM) and succinate (20mM), respectively. OCR was measured by an oxygen micro-respiration electrode and expressed as μM O_2_/min/10^6^ cells. Each is representative of at least three experiments. Panels F-G describe the activity of F0-F1 ATP synthase of CT26 cell lines, measured by luminometric analysis, after the addition of 10 mM pyruvate + 5 mM malate or 20 mM succinate. Panels H-I show the OCR and ATP synthesis in CT26 cells in the presence of pyruvate (10mM)/malate (5mM) and pre-incubated with Rotenone. Each is representative of at least six experiments. P value was calculated using unpaired t-test with Welch's correction. *: P<0.05.

Proteomic and genomic analyses did not show a clear effect of STS in up- regulating OXPHOS although different sets of respiratory enzymes were elevated after STS or OXP or STS+OXP (Supplementary Figure 2A-B). Clear changes were instead observed when measuring enzymatic activities of respiratory complexes, oxygen consumption and ATP: STS up-regulated Complex I and IV (Figure 4A and C) without affecting Complex II activity (Figure 4B). Consistent with this effect, a significant increase in O_2_consumption rate (OCR), indicative of an increased oxidative metabolism, was observed (Figure 4D and E). This corresponded to a significant reduction of ATP synthesis (Figure 4F and G). Accordingly, the ATP/AMP ratio, a good indicator of cellular energy charge, was dramatically reduced by the two STS settings (Supplementary Figure 2C).

In order to confirm the stimulatory effect of STS on Complex I-activity, we pre-incubated CT26 cells with Rotenone, a Complex I inhibitor. In the presence of Rotenone, none of the treatments caused a significant OCR increase (Figure 4H) or ATP synthesis reduction (Figure 4I).

**Figure 5:**
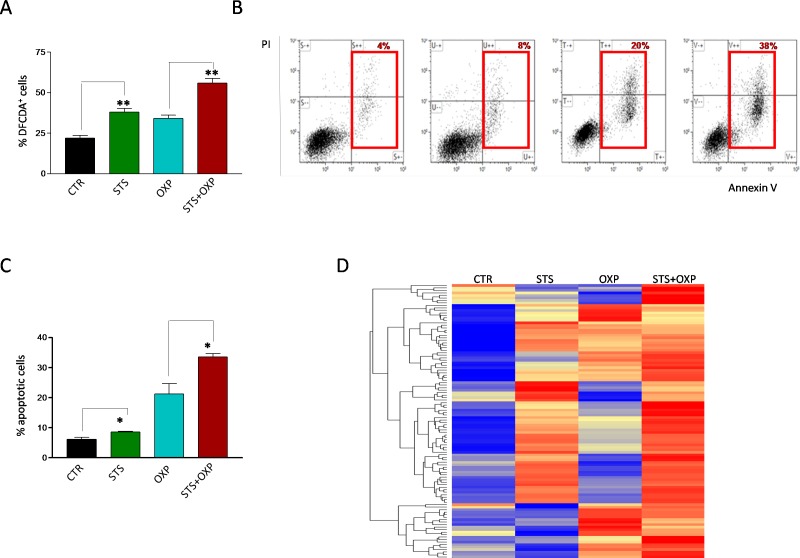
Effect of STS in combination with chemotherapy on reactive oxygen species production and apoptosis of colon carcinoma cells Panel A shows the oxidation state of the dye Dichlorodihydrofluoresceindiacetate (DCFDA) as a method to assess the level of reactive oxygen species (ROS) in CT26 colon carcinoma cells, undergoing STS (48 hours) or under standard conditions, and treated with or without 100μM oxaliplatin (OXP) for 24 hours. Results are expressed as mean of the percentage of DCFDA positive cells from three different experiments. Panel B shows a representative dot plot of CT26 cells, undergoing STS or under standard conditions, treated with or without OXP (100 μM) and stained with FITC Annexin V and propidium iodide (PI). Red squares indicate the percentage of Annexin^+^/PI^−^ and Annexin^+^/PI^+^ apoptotic cells (early and late apoptosis). Panel C shows the percentage of Annexin^+^/PI^−^ and Annexin^+^/PI^+^ apoptotic CT26 cells expressed as mean value ± SD from three different experiments performed. P value was calculated using Unpaired t test with Welch's correction. *: P<0.05; **: P<0.01. Panel D shows a representative heat map of proteins annotated by Gene Ontology as involved in apoptosis. Data are expressed as ratios over mean values for the four conditions (STS, OXP, STS+OXP; red = expression above mean, yellow = expression at mean; blue = expression below mean).

Increased activity of the mitochondrial respiratory chain with a decreased ATP production suggests a possible increase in superoxide generation caused by electron leakage, possibly at complex I and III [22]. Indeed, STS and OXP markedly increased ROS generation in CT26 cells and STS+OXP further exacerbated ROS production (Figure 5A). These profound metabolic alterations, characterized by reduced ATP biosynthesis and increased ROS production, are likely responsible for the additive effect of STS+OXP in triggering apoptosis in CT26 cells (Figure 5B-C).

This response was confirmed by proteomic analyses (Figure 5D) documenting an apoptotic signature obtained by hierarchical clustering of a 110 apoptotic proteins (GO:0006915) [23]. STS, OXP and especially STS+OXP induced the expression of many pro-apoptotic proteins (Figure 5D). In summary, these results strongly support the effect of STS on reducing glycolysis and lactate production and increasing respiration at Complexes I-IV resulting in superoxide production/oxidative stress but in reduced ATP generation.

## DISCUSSION

Our results indicate that STS, defined as a water only diet lasting 2 days in mice or severe serum plus glucose restriction *in vitro*, causes a profound metabolic shift and promotes an anti-Warburg effect in colon cancer cells. We show that STS *in vitro* down-regulates glycolysis and glutaminolysis and increases OXPHOS and OCR, reduces ATP synthesis and increased ROS production. This latter effect is presumably associated with the induction of apoptosis. Chemotherapy increased this toxicity further by inducing both Complex I and Complex II-dependent respiration, leading to elevated toxic oxygen species generation but reduced ATP generation and death.

Cancer cells alter their metabolism in order to satisfy their bioenergetic and biosynthetic requirements, generating a peculiar metabolic pattern characterized by high aerobic glycolysis, fatty acid synthesis and glutamine metabolism [1]. Aerobic glycolysis produces higher ATP and NADH levels [24, 25] which may contribute to chemoresistance through increased drug efflux from the cell, enhanced DNA repair, dysregulation of growth factor signaling and high expression of survival pathways or anti-apoptotic genes [26]. For these reasons, the combination of chemotherapy and agents able to affect the ability of cancer cells to adopt alternate metabolic strategies and survive represents a promising strategy to overcome drug resistance and improve chemotherapeutic index [27]. Therapies targeting cancer metabolism such as calorie restriction and ketogenic diet [28, 29] can modify the metabolic state of the whole organism but also that of the tumor cells. Part of the effects of STS could be achieved by agents that target metabolic enzymes including glucose transporters, hexokinase, pyruvate kinase M2, lactate dehydrogenase, lactate transporter, and glutaminase [4]. However, STS provides two advantages: 1) in both mice and humans it causes virtually no severe side effects since it differentially affects normal and cancer cells, 2) it alters the function of hundreds of proteins and enzymes thus increasing the chance of affecting a pathway required for tumor cell survival, particularly under the already hostile environment generated by chemotherapy.

Multiple cycles of STS have been shown to protect normal cells against chemotoxicity and to increase the effectiveness of chemotherapeutic drugs against the survival and progression of various tumor types [16, 30]. However, whether STS sensitizes tumor cells to chemotherapy by affecting their metabolism is presently unknown. In agreement with previous reports [17, 18, 21], our present results demonstrate that STS, especially in combination with chemotherapy, delays the *in vivo* and *in vitro* growth of colon carcinoma cells. We show that STS significantly reduces cancer glucose consumption leading to a transient arrest in cancer progression followed by a rebound phase after re-feeding. By contrast, STS+OXP causes a more severe metabolic impairment and a long-lasting decrease in cancer growth. Both observations confirm the relationship between glucose consumption and proliferation rate in cancer.

The profound metabolic alterations observed in tumor cells are often related to induction of PI3K-AKT signaling, which plays a pivotal role in glucose metabolism and cancer growth control [1]. Phosphorylated AKT has been shown to: i) up-regulate the expression of glucose transporters, ii) enhance glucose capture by HK and iii) to stimulate PFK [2]. Our study indicates that STS down-regulates PI3K, AKT, and PDK1 phosphorylation in CT26 colon carcinoma cells. In contrast, the expression of PTEN, which negatively regulates PI3K-AKT signaling, is significantly induced by STS.

Colon carcinoma cells cultured under starvation conditions exhibit a significant reduction of glucose avidity and consumption. GLUT1, a member of the GLUT (SLC2A) family of membrane transporters that mediates glucose import, is frequently overexpressed in many cancers. Its inhibition can overcome tumor chemoresistance and improve the therapeutic effect of chemodrugs [31-33]. In CT26 cells, glucose intake is known to be mainly dependent on GLUT1 and GLUT2 whose expression levels are dramatically down-regulated by STS. Glucose consumption and the start of the glycolytic process in cancer depends on HK II whose catalytic function is largely empowered by its p-AKT-dependent binding to the outer mitochondrial membrane [34]. We show that STS hampers this association which may limit the preferential access of HK II to ATP for glucose-6-phosphate production. STS also appears to inhibit PFK,PK and LDH activity, which will directly inhibit the rate-limiting steps of glycolytic flux. In agreement with the results above, STS dramatically reduces the lactate released by CT26 cells, likely due to the concomitant down-regulation of MCT1/Scl16a1 and CD147 (data not shown). The acidification of the tumor microenvironment due to high levels of exported lactate can hamper antitumor immune responses and favor tumor metastasis [35, 36]. Also, intracellular lactate may be converted to pyruvate by LDH and used as a nutrient source for oxidative metabolism and/or gluconeogenesis. Marked increase of LDHA, MCT1/4 and CD147 levels correlate with cancer progression and drug resistance [37-39] and the targeting of LDHA and MCT has been proposed as a cancer therapy [40-44]. Thus, the effect of STS on glycolysis and lactate production alone, could cause a major effect on cancer progression, even independently of its effects on mitochondria.

The inhibition of glucose utilization is accompanied by a down-regulatory effect of STS on the glutamine transporters Slc1a5 and glutaminase, which catalyze glutamine catabolism and provide cancer cells with bio-synthetic precursors for aminoacids and DNA synthesis [45-47].

**Figure 6:**
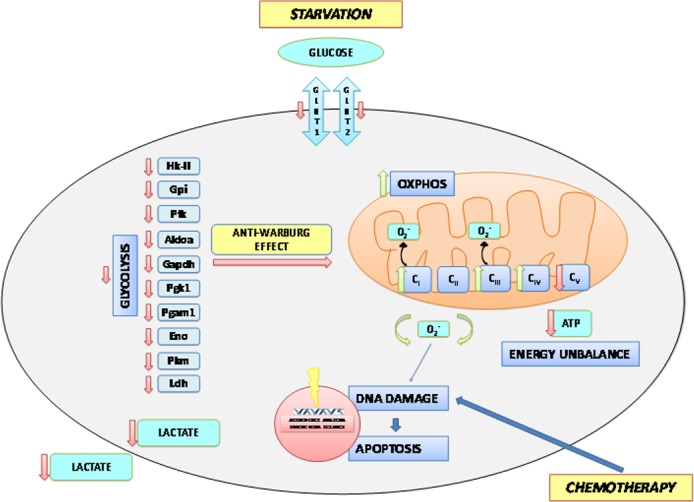
A Model of Short term starvation effects on the metabolism of the tumor cell Starvation in terms of reduced availability of glucose and aminoacids in the extracellular space determines a reduced expression of the glucose transporters Glut1 and Glut2 and leads to a general reduction in the glycolytic rate through the down-regulation of gene and protein expression as well as activity of almost all enzymes of the glycolytic chain. As a consequence, the production and secretion of lactate is reduced, with potential effects on the tumor microenvironment. This anti-Warburg effect is also accompanied by increased activities of complexes I, III and IV of the respiratory chain. However, uncoupling of respiration leads to the production of increased amounts of free oxygen radicals that determine an enhanced oxidative damage to the nuclear DNA. This latter event is responsible for the induction of apoptotic cell death. Uncoupling also causes a reduced activity of complex V of the respiratory chain resulting in ATP deficiency which makes tumor cell more vulnerable to the chemotherapeutic insult.

As a metabolic counterpart of glycolysis inhibition, STS induces ROS levels and apoptosis, probably as the result of a futile enhancement of mitochondrial complex activity uncoupled from ATP synthesis. Mitochondrial OXPHOS is a major cellular source of ROS, mainly through the activity of complex I and III. The most likely explanation for the increase in Complex I and IV activities, accompanied by increased OCR but reduced ATP generation and elevated ROS production after STS, OXP or their combination is that the cancer cells are not able to synthesize ATP at the rate dictated by oxygen consumption therefore increasing the electron leakage at Complexes I and III to form superoxide [1, 13, 22] (Figure 6). Higher superoxide and lower ATP could then promote cell death in part through ROS-induced apoptosis [12, 48, 49]. On the other hand, OXP, as a chemotherapeutic agent, provokes DNA damage-dependent apoptosis. Hence, the apoptotic activities induced by STS and chemotherapy complement each other. In conclusion, the present study indicates that the combination of STS cycles and chemotherapy causes a major enhancement of chemotherapeutic index, in part by promoting an anti-Warburg effect characterized by reduced glycolysis, reduced lactate generation, and increased oxidative phosphorylation with lower ATP synthesis but higher ROS generation and apoptosis.

## MATERIALS AND METHODS

### Cell lines and culture conditions

Tumor cell lines were cultured in DMEM medium (Euroclone, Milan, Italy) supplemented with 1% L-glutamine, penicillin/streptomycin, nonessential amino acids and 10% fetal bovine serum (FBS) (all from Sigma Aldrich, Milan, Italy) (complete medium). All treatments were performed at 37^o^C under 5% CO_2_. Glucose restriction was done by incubating cells in glucose free DMEM (Invitrogen, Monza, Italy) supplemented with either low glucose (0.5 g/L) or normal glucose (1.0 g/L) for 48 hours Serum restriction was done by incubating cells in DMEM/F12 with either 10% or 1% FBS for 48 hours. Oxaliplatin (OXP, Hospira, Naples, Italy) treatment was performed 24 hours after STS in a concentration range between 10-100 μM. Optimum drug doses were determined for each individual cell line.

### Cell viability, proliferation and apoptosis assays

Colon cancer cell lines were plated in 12-well plates (Falcon, Becton Dickinson, Le Pont de Claix, France) (CT26 and HCT 116 1×10^5^, HT-29 2×10^5^, cells per well) in complete medium. After 24 hours media were switched to starvation (0.5 g/L glucose + 1% FBS) or control (1 g/L glucose + 10% FBS) and 48 hours later cells were harvested and the number was assessed by Trypan blue (Sigma Aldrich) exclusion. To asses proliferation, cancer cells were labeled with 20 μM CarboxyfluoresceinSuccinimidyl ester (CFSE) (Invitrogen, Milano, Italy) for 15 minutes at 37°C and treated as above described. Then, cells were acquired with a Gallios cytometer.

### Immunofluorescence analysis

CT26 cells were incubated with MitoTracker probe (Life Technologies Italia, Monza, Italy) and treated as previously described [50]. Coverslips were incubated overnight with rabbit anti-HKI (C35C4) or anti-HKII (C64G5) primary antibodies (Cell Signaling Technologies, Leiden, The Netherlands). Specific staining was visualized with a goat anti-Rabbit Alexa Fluor 488 secondary antibody (Molecular Probe® Life Technology), washed and mounted using Prolong Gold anti-fading reagent (Life Technologies). Results were analyzed using an Olympus (Olympus Optical) laser-scanning microscope FV500 equipped with an Olympus IX81 inverted microscope and Argon ion 488 nm, He-Ne 543 nm, and He-Ne 633 nm lasers. Digital images were acquired through a PLAPO 60× objective, with the Fluoview 4.3b software program, sequentially as single trans-cellular optical sections. Spatial co-localization was analyzed by Image J 1.34f software (NIH).

### Western blotting

The antibodies used were as follows: anti-PTEN (Millipore), anti-Phospho-AKT (Ser473), anti-Phospho-PDK1, anti-PI3K, anti-HKII, anti-PFK1, anti-PKM2, anti-β-actin (all from Cell Signaling), and anti-GLUT1, GLUT2, GLUT3, GLUT4 (all from Cell Abcam, United Kingdom).

### Spectrophotometric enzymes assay

Glycolytic enzymes were assayed at room temperature on 25 μg of CT26 cells protein, in a double beam spectrophotometer (UNICAM UV2, Analytical S.n.c., Italy) as previously described [51]. The activity assay of the redox complexes I, II, III, IV was evaluated as described in [52, 53]. ATP and AMP were measured according to the enzyme coupling method described elsewhere [54].

### Oxygraphic measurements

O_2_ consumption was measured at 25 °C in a closed chamber, using a thermostatically controlled oxygraph apparatus equipped with amperometric electrode (Microrespiration, Unisense A/S, Århus, Denmark) as described [55]. In some experiments, CT26 cells were pre-incubated with 50 μM Rotenone (Sigma) 5 minutes before performing O_2_ measure.

### Bioluminescent luciferase ATP assay

Fifty micrograms of CT26 cell proteins were incubated in an appropriate buffer plus 5 mM pyruvate and 2.5 mM malate as described in [56] and ATP synthesis was then induced adding 0.1 mM ADP. ATP concentration was measured in a luminometer (Lumi-Scint, Bioscan) by the luciferin/luciferase chemiluminescent method [57]. In some experiments, CT26 cells were pre-incubated with 50 μM Rotenone (Sigma) 5 minutes before performing ATP measure. In all experiments the ATP synthesis rate was linear for the first 2 minutes.

### Proteomic analysis

The MS instrument was operated in data-dependent mode to automatically switch between full-scan MS and MS/MS acquisition. Survey full-scan MS spectra were acquired in the Orbitrap analyzer with resolution R = 60,000. The 20 most intense peptide ions with charge states ≥ 2 were sequentially isolated and fragmented by collision-induced dissociation (CID) in the LTQ mass spectrometer. Raw mass spectrometric data were analyzed using the MaxQuant pipeline [58]. Moreover quantification and data normalization were achieved by an intensity-based label-free approach using MaxQuant. Thanks to the implementation of maximal peptide ratio extraction and delayed normalization algorithms, the MaxLFQ program included in MaxQuant, provide accurate XIC-based quantification [59]. The statistical and the pathways analysis have been made with the freely available Perseus (www.perseusframework.org/Perseus_1.4.1.3.zip) and Cytoscape software respectively [60].

### Microarray

RNA was extracted from cell lines using RNeasy (Qiagen, Hilden, Germany). RNA quality was assessed in the BioAnalyzer (Agilent, St. Clara, CA). cDNA, ds-cDNA and cRNA synthesis was performed using the 3′ IVT Express Kit (Affymetrix, Santa Clara, CA, USA) according to the manufacturer's instructions. cRNAs were purified using the RNeasy Mini Kit (Qiagen), controlled by agarose gel electrophoresis and subjected to fragmentation for 35 min. at 94°C in fragmentation buffer (40mM Tris-acetate pH 8.1, 100mM CH_3_COOH, 30mM Mg(CH_3_COOH)2×4H2O). Hybridization, washing and staining were performed using the GeneAtlas® Hybridization, Wash, and Stain Kit for 3′ IVT Arrays (Affymetrix, St. Clara, CA).

### Mouse models

To establish a subcutaneous cancer mouse model, 6 week-old female BALB/c mice were injected subcutaneously in the lower back with 100 μL CT26 colon cancer cells resuspended in PBS at a density of 2×10^6^ cells/mL (2×10^5^ cells/mouse). Five days after tumor cell inoculum, when tumors were palpable, the animals were subdivided into four groups: “Controls” (N=7) with tumor and no treatment which were kept under standard conditions for the whole duration of the study; “STS” animals (N=7) that were submitted to 48-hour STS, “OXP” mice (N=7) treated with OXP for 24 hours, “STS+OXP” mice (N=7) submitted to both treatments. At the end of all treatments mice were submitted to micro-PET imaging. STS consisted in a complete food deprivation with free access to water. “STS” mice were individually housed in a clean new cage to reduce cannibalism and were monitored daily for weight loss and general behavior. “OXP” mice were inoculated intraperitoneally (ip) with OXP at 10 mg/Kg and housed in the presence of food. Tumor size was measured by caliper and tumor volume was calculated using the following equation: tumor volume (mm^3^) = (length × width × height) x π/6, expressing length, width and height in mm.

### Fluorodeoxyglucose uptake evaluation

Daily quality controls always documented adequate standards and, in particular, a radiochemical purity ≥ 98%. Labeling was performed by incubating 10^6^ cells with FDG according to a procedure validated in our laboratory [50, 61]. Immediately before the experiment, glucose free medium was added with two mL PBS containing FDG at a concentration of 37 KBq/mL. Tracer exposure was maintained for 60 minutes at 37°C. Thereafter, the uptake process was stopped by adding 4 ml of PBS before centrifugation at 450 g for 10 minutes. Supernatant was removed and cell pellet re-suspended in 1ml of saline buffer. Free and bound activities were thus simultaneously counted using a Packard Cobra II gamma counter (Packard, Meriden, CT) with a 10% energy window centered at 511KeV. FDG retention was measured as the ratio between bound and total radioactivity. In all cases, the labeling procedure did not affect cell viability as documented by Trypan blue staining.

### *In vivo* micro-PET

Mice were weighted and anesthesia was induced by ip administration of ketamine (100 mg/Kg) (IMALGERE 1000, Milan, Italy)/xylazine (10 mg/kg) (BIO98 Srl, Milan, Italy). Serum glucose level was tested and animals were positioned on the bed of a dedicated micro-PET system (Albira, Carestream Inc, US) whose two-ring configuration permits to cover the whole animal body in a single bed position. A dose of 3-4 MBq of FDG was then injected through a tail vein, soon after start of a list mode acquisition lasting 50 minutes.

### *In vivo* image processing

PET data were reconstructed using a maximal likelihood expectation maximization method (MLEM). An experienced observer, unaware of the experimental type of analyzed mouse, identified a volume of interest (VOI) in the left ventricular chamber. Then, the computer was asked to plot the time-concentration curve within this VOI throughout the whole acquisition to define tracer input function. Whole body FDG clearance (in ml x min^−1^) was calculated using the conventional stochastic approach as the ratio between injected dose and integral of input function from 0 to infinity, fitting the last 20 minutes with a mono-exponential function [62]. A further VOI was drawn over cancer lesion to measure maximal standardized uptake value (SUV), i.e. the most commonly accepted index of tissue FDG uptake, expressed as the fraction of injected tracer dose normalized for body weight.

Tumor glucose consumption was expressed in nM X min^−1^ X g^−1^ and was estimated in this last VOI according to Gjedde-Patlak [63] graphical analysis by using the routine of a dedicated software (PMOD, Zurich, Switzerland). Briefly, the software utilizes the input function to transform the original tissue activity measurements by fitting the data in each voxel with the slope of the regression line defined by the model. In all cases, lumped constant value was set at 1.

### Statistical analysis

The *in vivo* data are presented as mean ± standard deviation (SD). For comparison between different groups, the Null hypothesis was tested by a single factor analysis of variance (ANOVA) for multiple groups. Statistical significance was considered for p values p<0.05.

For *in vitro* experiments, the statistical significance of differences between experimental and control groups was assessed by Unpaired t test with Welch's correction using GraphPad Prism 3.0 software (GraphPad Software, Inc, El Camino Real, San Diego, CA).

## SUPPLEMENTARY MATERIAL AND FIGURES



## References

[1] Vander Heiden MG, Cantley LC, Thompson CB (2009). Understanding the Warburg effect: the metabolic requirements of cell proliferation. Science.

[2] DeBerardinis RJ, Lum JJ, Hatzivassiliou G, Thompson CB (2008). The biology of cancer: metabolic reprogramming fuels cell growth and proliferation. Cell Metab.

[3] DeBerardinis RJ, Mancuso A, Daikhin E, Nissim I, Yudkoff M, Wehrli S, Thompson CB (2007). Beyond aerobic glycolysis: transformed cells can engage in glutamine metabolism that exceeds the requirement for protein and nucleotide synthesis. Proc Natl Acad Sci U S A.

[4] Zhao Y, Butler EB, Tan M (2013). Targeting cellular metabolism to improve cancer therapeutics. Cell Death Dis.

[5] Zhou M, Zhao Y, Ding Y, Liu H, Liu Z, Fodstad O, Riker AI, Kamarajugadda S, Lu J, Owen LB, Ledoux SP, Tan M (2010). Warburg effect in chemosensitivity: targeting lactate dehydrogenase-A re-sensitizes taxol-resistant cancer cells to taxol. Mol Cancer.

[6] Tamada M, Nagano O, Tateyama S, Ohmura M, Yae T, Ishimoto T, Sugihara E, Onishi N, Yamamoto T, Yanagawa H, Suematsu M, Saya H (2012). Modulation of glucose metabolism by CD44 contributes to antioxidant status and drug resistance in cancer cells. Cancer Res.

[7] Tome ME, Frye JB, Coyle DL, Jacobson EL, Samulitis BK, Dvorak K, Dorr RT, Briehl MM (2012). Lymphoma cells with increased anti-oxidant defenses acquire chemoresistance. Exp Ther Med.

[8] Nogueira V, Hay N (2013). Molecular pathways: reactive oxygen species homeostasis in cancer cells and implications for cancer therapy. Clin Cancer Res.

[9] Tennant DA, Duran RV, Gottlieb E (2010). Targeting metabolic transformation for cancer therapy. Nat Rev Cancer.

[10] Madhok BM, Yeluri S, Perry SL, Hughes TA, Jayne DG (2010). Dichloroacetate induces apoptosis and cell-cycle arrest in colorectal cancer cells. Br J Cancer.

[11] Wise DR, Thompson CB (2010). Glutamine addiction: a new therapeutic target in cancer. Trends Biochem Sci.

[12] Hamanaka RB, Chandel NS (2010). Mitochondrial reactive oxygen species regulate cellular signaling and dictate biological outcomes. Trends Biochem Sci.

[13] Brand MD, Affourtit C, Esteves TC, Green K, Lambert AJ, Miwa S, Pakay JL, Parker N (2004). Mitochondrial superoxide: production, biological effects, and activation of uncoupling proteins. Free Radic Biol Med.

[14] Trachootham D, Alexandre J, Huang P (2009). Targeting cancer cells by ROS-mediated mechanisms: a radical therapeutic approach?. Nat Rev Drug Discov.

[15] Diehn M, Cho RW, Lobo NA, Kalisky T, Dorie MJ, Kulp AN, Qian D, Lam JS, Ailles LE, Wong M, Joshua B, Kaplan MJ, Wapnir I (2009). Association of reactive oxygen species levels and radioresistance in cancer stem cells. Nature.

[16] Raffaghello L, Lee C, Safdie FM, Wei M, Madia F, Bianchi G, Longo VD (2008). Starvation-dependent differential stress resistance protects normal but not cancer cells against high-dose chemotherapy. Proc Natl Acad Sci U S A.

[17] Lee C, Raffaghello L, Brandhorst S, Safdie FM, Bianchi G, Martin-Montalvo A, Pistoia V, Wei M, Hwang S, Merlino A, Emionite L, de Cabo R, Longo VD (2012). Fasting cycles retard growth of tumors and sensitize a range of cancer cell types to chemotherapy. Sci Transl Med.

[18] Safdie F, Brandhorst S, Wei M, Wang W, Lee C, Hwang S, Conti PS, Chen TC, Longo VD (2012). Fasting enhances the response of glioma to chemo- and radiotherapy. PLoS One.

[19] Safdie FM, Dorff T, Quinn D, Fontana L, Wei M, Lee C, Cohen P, Longo VD (2009). Fasting and cancer treatment in humans: A case series report. Aging (Albany NY).

[20] Cheng CW, Adams GB, Perin L, Wei M, Zhou X, Lam BS, Da Sacco S, Mirisola M, Quinn DI, Dorff TB, Kopchick JJ, Longo VD (2014). Prolonged fasting reduces IGF-1/PKA to promote hematopoietic-stem-cell-based regeneration and reverse immunosuppression. Cell Stem Cell.

[21] Shi Y, Felley-Bosco E, Marti TM, Orlowski K, Pruschy M, Stahel RA (2012). Starvation-induced activation of ATM/Chk2/p53 signaling sensitizes cancer cells to cisplatin. BMC Cancer.

[22] Venditti P, Di Stefano L, Di Meo S (2013). Mitochondrial metabolism of reactive oxygen species. Mitochondrion.

[23] Dimmer EC, Huntley RP, Alam-Faruque Y, Sawford T, O'Donovan C, Martin MJ, Bely B, Browne P, Mun Chan W, Eberhardt R, Gardner M, Laiho K, Legge D (2012). The UniProt-GO Annotation database in 2011. Nucleic Acids Res.

[24] Fanciulli M, Bruno T, Giovannelli A, Gentile FP, Di Padova M, Rubiu O, Floridi A (2000). Energy metabolism of human LoVo colon carcinoma cells: correlation to drug resistance and influence of lonidamine. Clin Cancer Res.

[25] Zhou Y, Tozzi F, Chen J, Fan F, Xia L, Wang J, Gao G, Zhang A, Xia X, Brasher H, Widger W, Ellis LM, Weihua Z (2012). Intracellular ATP levels are a pivotal determinant of chemoresistance in colon cancer cells. Cancer Res.

[26] Longley DB, Johnston PG (2005). Molecular mechanisms of drug resistance. J Pathol.

[27] Butler EB, Zhao Y, Munoz-Pinedo C, Lu J, Tan M (2013). Stalling the engine of resistance: targeting cancer metabolism to overcome therapeutic resistance. Cancer Res.

[28] Kalaany NY, Sabatini DM (2009). Tumours with PI3K activation are resistant to dietary restriction. Nature.

[29] Champ CE, Palmer JD, Volek JS, Werner-Wasik M, Andrews DW, Evans JJ, Glass J, Kim L, Shi W (2014). Targeting metabolism with a ketogenic diet during the treatment of glioblastoma multiforme. J Neurooncol.

[30] Lee C, Safdie FM, Raffaghello L, Wei M, Madia F, Parrella E, Hwang D, Cohen P, Bianchi G, Longo VD (2010). Reduced levels of IGF-I mediate differential protection of normal and cancer cells in response to fasting and improve chemotherapeutic index. Cancer Res.

[31] Cao X, Fang L, Gibbs S, Huang Y, Dai Z, Wen P, Zheng X, Sadee W, Sun D (2007). Glucose uptake inhibitor sensitizes cancer cells to daunorubicin and overcomes drug resistance in hypoxia. Cancer Chemother Pharmacol.

[32] Liu Y, Cao Y, Zhang W, Bergmeier S, Qian Y, Akbar H, Colvin R, Ding J, Tong L, Wu S, Hines J, Chen X (2012). A small-molecule inhibitor of glucose transporter 1 downregulates glycolysis, induces cell-cycle arrest, and inhibits cancer cell growth *in vitro* and *in vivo*. Mol Cancer Ther.

[33] Chan DA, Sutphin PD, Nguyen P, Turcotte S, Lai EW, Banh A, Reynolds GE, Chi JT, Wu J, Solow-Cordero DE, Bonnet M, Flanagan JU, Bouley DM (2011). Targeting GLUT1 and the Warburg effect in renal cell carcinoma by chemical synthetic lethality. Sci Transl Med.

[34] Robey RB, Hay N (2006). Mitochondrial hexokinases, novel mediators of the antiapoptotic effects of growth factors and Akt. Oncogene.

[35] Swietach P, Vaughan-Jones RD, Harris AL (2007). Regulation of tumor pH and the role of carbonic anhydrase 9. Cancer Metastasis Rev.

[36] Fischer K, Hoffmann P, Voelkl S, Meidenbauer N, Ammer J, Edinger M, Gottfried E, Schwarz S, Rothe G, Hoves S, Renner K, Timischl B, Mackensen A (2007). Inhibitory effect of tumor cell-derived lactic acid on human T cells. Blood.

[37] Hao J, Chen H, Madigan MC, Cozzi PJ, Beretov J, Xiao W, Delprado WJ, Russell PJ, Li Y (2010). Co-expression of CD147 (EMMPRIN), CD44v3-10, MDR1 and monocarboxylate transporters is associated with prostate cancer drug resistance and progression. Br J Cancer.

[38] Chen H, Wang L, Beretov J, Hao J, Xiao W, Li Y (2010). Co-expression of CD147/EMMPRIN with monocarboxylate transporters and multiple drug resistance proteins is associated with epithelial ovarian cancer progression. Clin Exp Metastasis.

[39] Kang MJ, Kim HP, Lee KS, Yoo YD, Kwon YT, Kim KM, Kim TY, Yi EC (2013). Proteomic analysis reveals that CD147/EMMPRIN confers chemoresistance in cancer stem cell-like cells. Proteomics.

[40] Fang J, Quinones QJ, Holman TL, Morowitz MJ, Wang Q, Zhao H, Sivo F, Maris JM, Wahl ML (2006). The H+-linked monocarboxylate transporter (MCT1/SLC16A1): a potential therapeutic target for high-risk neuroblastoma. Mol Pharmacol.

[41] Gerlinger M, Santos CR, Spencer-Dene B, Martinez P, Endesfelder D, Burrell RA, Vetter M, Jiang M, Saunders RE, Kelly G, Dykema K, Rioux-Leclercq N, Stamp G (2012). Genome-wide RNA interference analysis of renal carcinoma survival regulators identifies MCT4 as a Warburg effect metabolic target. J Pathol.

[42] Doherty JR, Yang C, Scott KE, Cameron MD, Fallahi M, Li W, Hall MA, Amelio AL, Mishra JK, Li F, Tortosa M, Genau HM, Rounbehler RJ (2014). Blocking Lactate Export by Inhibiting the Myc Target MCT1 Disables Glycolysis and Glutathione Synthesis. Cancer Res.

[43] Le Floch R, Chiche J, Marchiq I, Naiken T, Ilc K, Murray CM, Critchlow SE, Roux D, Simon MP, Pouyssegur J (2011). CD147 subunit of lactate/H+ symporters MCT1 and hypoxia-inducible MCT4 is critical for energetics and growth of glycolytic tumors. Proc Natl Acad Sci U S A.

[44] Baba M, Inoue M, Itoh K, Nishizawa Y (2008). Blocking CD147 induces cell death in cancer cells through impairment of glycolytic energy metabolism. Biochem Biophys Res Commun.

[45] Todorova VK, Kaufmann Y, Luo S, Klimberg VS (2011). Tamoxifen and raloxifene suppress the proliferation of estrogen receptor-negative cells through inhibition of glutamine uptake. Cancer Chemother Pharmacol.

[46] Karunakaran S, Ramachandran S, Coothankandaswamy V, Elangovan S, Babu E, Periyasamy-Thandavan S, Gurav A, Gnanaprakasam JP, Singh N, Schoenlein PV, Prasad PD, Thangaraju M, Ganapathy V (2011). SLC6A14 (ATB0,+) protein, a highly concentrative and broad specific amino acid transporter, is a novel and effective drug target for treatment of estrogen receptor-positive breast cancer. J Biol Chem.

[47] Wang JB, Erickson JW, Fuji R, Ramachandran S, Gao P, Dinavahi R, Wilson KF, Ambrosio AL, Dias SM, Dang CV, Cerione RA (2010). Targeting mitochondrial glutaminase activity inhibits oncogenic transformation. Cancer Cell.

[48] Brenner C, Grimm S (2006). The permeability transition pore complex in cancer cell death. Oncogene.

[49] Kamata H, Honda S, Maeda S, Chang L, Hirata H, Karin M (2005). Reactive oxygen species promote TNFalpha-induced death and sustained JNK activation by inhibiting MAP kinase phosphatases. Cell.

[50] Salani B, Marini C, Rio AD, Ravera S, Massollo M, Orengo AM, Amaro A, Passalacqua M, Maffioli S, Pfeffer U, Cordera R, Maggi D, Sambuceti G (2013). Metformin impairs glucose consumption and survival in Calu-1 cells by direct inhibition of hexokinase-II. Sci Rep.

[51] Ravera S, Bartolucci M, Calzia D, Aluigi MG, Ramoino P, Morelli A, Panfoli I (2013). Tricarboxylic acid cycle-sustained oxidative phosphorylation in isolated myelin vesicles. Biochimie.

[52] Sottocasa GL, Kuylenstierna B, Ernster L, Bergstrand A (1967). An electron-transport system associated with the outer membrane of liver mitochondria. A biochemical and morphological study. J Cell Biol.

[53] Baracca A, Sgarbi G, Solaini G, Lenaz G (2003). Rhodamine 123 as a probe of mitochondrial membrane potential: evaluation of proton flux through F(0) during ATP synthesis. Biochim Biophys Acta.

[54] Ravera S, Vaccaro D, Cuccarolo P, Columbaro M, Capanni C, Bartolucci M, Panfoli I, Morelli A, Dufour C, Cappelli E, Degan P Mitochondrial respiratory chain Complex I defects in Fanconi anemia complementation group A. Biochimie.

[55] Ravera S, Panfoli I, Calzia D, Aluigi MG, Bianchini P, Diaspro A, Mancardi G, Morelli A (2009). Evidence for aerobic ATP synthesis in isolated myelin vesicles. Int J Biochem Cell Biol.

[56] Ravera S, Aluigi MG, Calzia D, Ramoino P, Morelli A, Panfoli I (2011). Evidence for ectopic aerobic ATP production on C6 glioma cell plasma membrane. Cell Mol Neurobiol.

[57] Ravera S, Panfoli I, Aluigi MG, Calzia D, Morelli A (2011). Characterization of Myelin Sheath F(o)F(1)-ATP synthase and its regulation by IF(1). Cell Biochem Biophys.

[58] Cox J, Mann M (2008). MaxQuant enables high peptide identification rates, individualized p.p.b.-range mass accuracies and proteome-wide protein quantification. Nat Biotechnol.

[59] Cox J, Hein MY, Luber CA, Paron I, Nagaraj N, Mann M (2014). Accurate proteome-wide label-free quantification by delayed normalization and maximal peptide ratio extraction, termed MaxLFQ. Mol Cell Proteomics.

[60] Cline MS, Smoot M, Cerami E, Kuchinsky A, Landys N, Workman C, Christmas R, Avila-Campilo I, Creech M, Gross B, Hanspers K, Isserlin R, Kelley R (2007). Integration of biological networks and gene expression data using Cytoscape. Nat Protoc.

[61] Wurth R, Pattarozzi A, Gatti M, Bajetto A, Corsaro A, Parodi A, Sirito R, Massollo M, Marini C, Zona G, Fenoglio D, Sambuceti G, Filaci G (2013). Metformin selectively affects human glioblastoma tumor-initiating cell viability: A role for metformin-induced inhibition of Akt. Cell Cycle.

[62] Iozzo P, Gastaldelli A, Jarvisalo MJ, Kiss J, Borra R, Buzzigoli E, Viljanen A, Naum G, Viljanen T, Oikonen V, Knuuti J, Savunen T, Salvadori PA (2006). 18F-FDG assessment of glucose disposal and production rates during fasting and insulin stimulation: a validation study. J Nucl Med.

[63] Patlak CS, Blasberg RG, Fenstermacher JD (1983). Graphical evaluation of blood-to-brain transfer constants from multiple-time uptake data. J Cereb Blood Flow Metab.

