# Supplementary material for: Fasting induces anti-Warburg effect that increases respiration but reduces ATP-synthesis to promote apoptosis in colon cancer models
Source: Oncotarget. 2015 Mar 18;6(14):11806–19. doi: 10.18632/oncotarget.3688 (PMC4494906; doi:10.18632/oncotarget.3688)
Supplement: Supplementary file 1 [file oncotarget-06-11806-s001.pdf]

Fasting induces anti-Warburg effect that increases respiration but reduces ATP-synthesis to promote apoptosis in colon cancer models

Supplementary Material

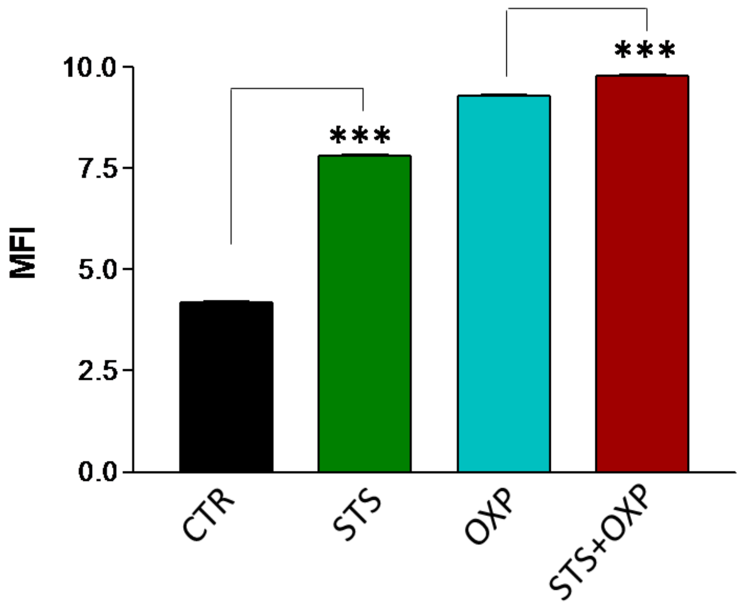

Supplementary Figure 1: Effects of starvation in combination with chemotherapy on proliferation of CT26 colon carcinoma cell lines.

CSFE labeled CT26 colon carcinoma cells were cultured in normal (1.0 g/L glucose + 10% FBS) or starved (0.5 g/L glucose + 1% FBS) conditions 48 hours. Cells were incubated with 40 μM oxaliplatin (OXP) for 24 hours. CSFE stained cells were analyzed by cytofluorimetry. MFI = mean fluorescence intensity.

A

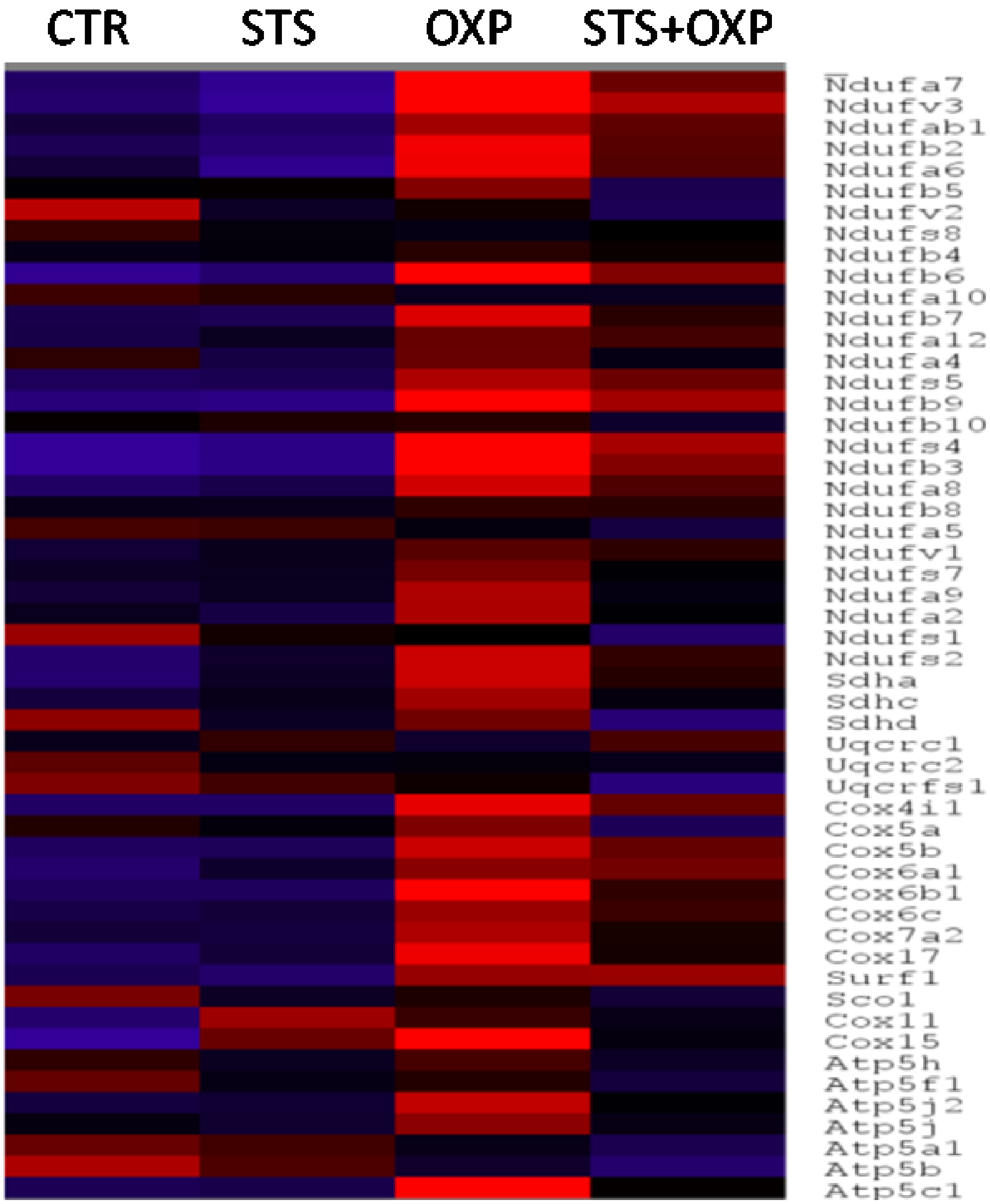

CTR
STS
OXP
STS+OXP
Ndufa7
Ndufv3
Ndufab1
Ndufb2
Ndufa6
Ndufb5
Ndufv2
Ndufs8
Ndufb4
Ndufb6
Ndufa10
Ndufb7
Ndufa12
Ndufa4
Ndufs5
Ndufb9
Ndufb10
Ndufs4
Ndufb3
Ndufa8
Ndufb8
Ndufa5
Ndufv1
Ndufs7
Ndufa9
Ndufa2
Ndufs1
Ndufs2
Sdha
Sdhc
Sdhd
Uqcrc1
Uqcrc2
Uqcrfs1
Cox4i1
Cox5a
Cox5b
Cox6a1
Cox6b1
Cox6c
Cox7a2
Cox17
Surf1
Sco1
Cox11
Cox15
Atp5h
Atp5f1
Atp5j2
Atp5j
Atp5a1
Atp5b
Atp5c1

B

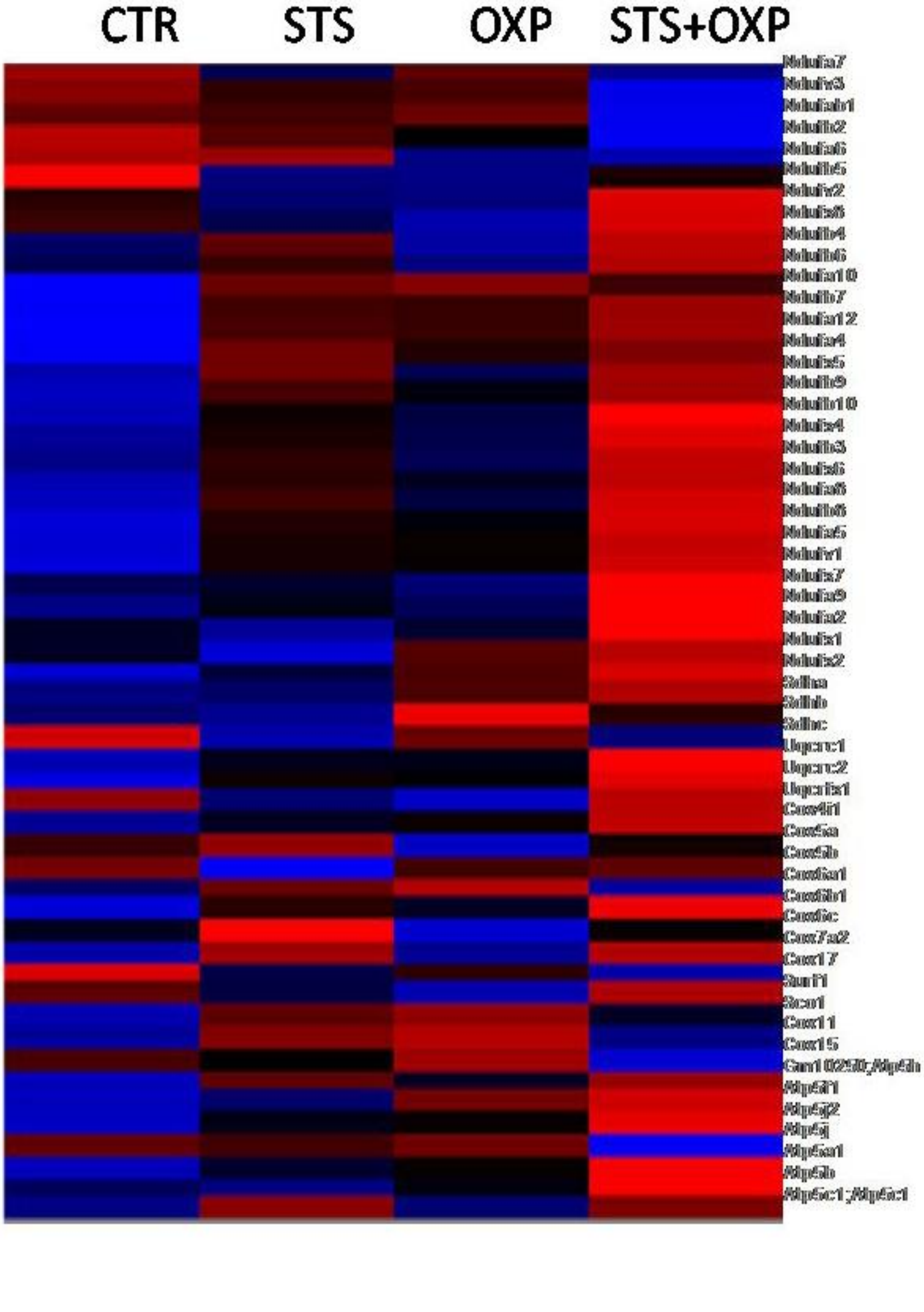
CTR
STS
OXP
STS+OXP
Ndufa7
Ndufv3
Ndufab1
Ndufb2
Ndufa6
Ndufb5
Ndufv2
Ndufs8
Ndufb4
Ndufb6
Ndufa10
Ndufb7
Ndufa12
Ndufa4
Ndufs5
Ndufb9
Ndufb10
Ndufs4
Ndufb3
Ndufs6
Ndufa8
Ndufb8
Ndufa5
Ndufv1
Ndufs7
Ndufa9
Ndufa2
Ndufs1
Ndufs2
Sdha
Sdhb
Sdhc
Uqcrc1
Uqcrc2
Uqcrfs1
Cox4i1
Cox5a
Cox5b
Cox6a1
Cox6b1
Cox6c
Cox7a2
Cox17
Surf1
Sco1
Cox11
Cox15
Gm10250;Atp5h
Atp5f1
Atp5j2
Atp5j
Atp5a1
Atp5b
Atp5c1;Atp5c1

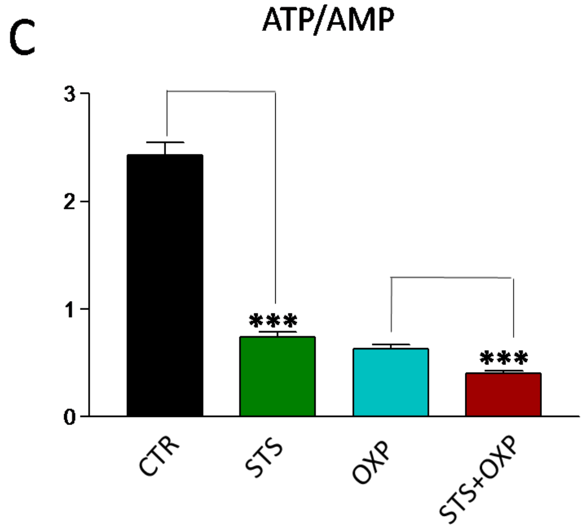

Supplementary Figure 2: Expression of oxidative phosphorylation enzymes and energy charge in response to starvation and chemotherapy in colon carcinoma cell lines

CT26 colon carcinoma cells were starved or not and treated with or without 40 μM oxaliplatin (OXP) for 24 hours. For protein expression analysis (Panel A) cells were lysed and injected in nanoscale high-performance liquid chromatography system connected to a hybrid linear trap quadrupole (LTQ) Orbitrap mass spectrometer. Proteins were identified and quantified using MaxQuant pipeline. For gene expression analysis (Panel B) RNA was extracted from CT26 cells and analyzed by microarray hybridization. Representative expression heatmaps of proteins (Panel A) and genes (Panel B) involved in oxidative phosphorylation are shown.Data are expressed as ratios over mean values for the four conditions (STS, OXP, STS+OXP; red = expression above mean, black = expression at mean; blue = expression below mean)

Panel C shows the ATP/AMP ratio evaluated by spectrometry in CT26, treated with or without STS, OXP or STS + OXP. Data are expressed as the mean value ± SD of ATP/AMP ratio.
